# Long-Term Engagement With a Mobile Self-Management System for People With Type 2 Diabetes

**DOI:** 10.2196/mhealth.2432

**Published:** 2013-03-27

**Authors:** Naoe Tatara, Eirik Årsand, Stein Olav Skrøvseth, Gunnar Hartvigsen

**Affiliations:** ^1^Norwegian Centre for Integrated Care and TelemedicineUniversity Hospital of North NorwayTromsøNorway; ^2^Department of Computer ScienceUniversity of TromsøTromsøNorway; ^3^Design of Information Systems GroupDepartment of InformaticsUniversity of OsloOsloNorway

**Keywords:** Type 2 diabetes, self-management, user-involved design process, mobile phone, usage, usability, mHealth

## Abstract

**Background:**

In a growing number of intervention studies, mobile phones are used to support self-management of people with Type 2 diabetes mellitus (T2DM). However, it is difficult to establish knowledge about factors associated with intervention effects, due to considerable differences in research designs and outcome measures as well as a lack of detailed information about participants’ engagement with the intervention tool.

**Objective:**

To contribute toward accumulating knowledge about factors associated with usage and usability of a mobile self-management application over time through a thorough analysis of multiple types of investigation on each participant’s engagement.

**Methods:**

The Few Touch application is a mobile-phone–based self-management tool for patients with T2DM. Twelve patients with T2DM who have been actively involved in the system design used the Few Touch application in a real-life setting from September 2008 until October 2009. During this period, questionnaires and semistructured interviews were conducted. Recorded data were analyzed to investigate usage trends and patterns. Transcripts from interviews were thematically analyzed, and the results were further analyzed in relation to the questionnaire answers and the usage trends and patterns.

**Results:**

The Few Touch application served as a flexible learning tool for the participants, responsive to their spontaneous needs, as well as supporting regular self-monitoring. A significantly decreasing (*P*<.05) usage trend was observed among 10 out of the 12 participants, though the magnitude of the decrease varied widely. Having achieved a sense of mastery over diabetes and experiences of problems were identified as reasons for declining motivation to continue using the application. Some of the problems stemmed from difficulties in integrating the use of the application into each participant’s everyday life and needs, although the design concepts were developed in the process where the participants were involved. The following factors were identified as associated with usability and/or usage over time: Integration with everyday life; automation; balance between accuracy and meaningfulness of data with manual entry; intuitive and informative feedback; and rich learning materials, especially about foods.

**Conclusion:**

Many grounded design implications were identified through a thorough analysis of results from multiple types of investigations obtained through a year-long field trial of the Few Touch application. The study showed the importance and value of involving patient-users in a long-term trial of a tool to identify factors influencing usage and usability over time. In addition, the study confirmed the importance of detailed analyses of each participant’s usage of the provided tool for better understanding of participants’ engagement over time.

## Introduction

For effective medical care of chronic illness, such as Type 2 diabetes mellitus (T2DM), adequate and sustainable self-management initiated by patients is important [1-3]. Nevertheless, poor adherence to T2DM treatment is common [4]. Mobile phones have been considered promising intervention platforms to support self-management of lifestyle-related diseases in general because of their pervasiveness and ubiquity [5]. Especially with the emergence of smartphones, the number of mobile self-management tools for diabetes available both commercially and free of charge is rapidly increasing [6-8]. Reflecting this situation, a growing number of studies report interventions using mobile phones and development projects of mobile self-management tools for people with diabetes [9-11]. However, considerable differences in research designs and outcome measures make it difficult to conduct rigorous meta-analysis of the findings [12,13]. In addition, recent reviews [11,14,15] point out a lack of focus on detailed reporting on participants’ long-term engagement with the intervention tools.

We recently conducted a literature review based on search criteria used previously [9] that cover more publication channels and more types of mobile terminals than the criteria used in the above-mentioned reviews do [11,14,15]. Our review also revealed considerable differences among the studies in terms of the level of detail regarding reports on participants’ engagement with the intervention tools over time. In two studies [16,17], two groups with different intervention conditions were compared in terms of change in average level of engagement among the participants with the passage of time, but differences between the participants in each group were not explained. Ten studies [18-27] identified differences in the level of engagement between the participants, but only three of them [18-20] reported how their level of engagement changed over time and the reasons for attrition of engagement. Two [26,27] of the 10 studies however reported individual participants’ levels of engagement and qualitatively analyzed participants’ experience of the tool to identify factors associated with usage. Four studies [28-31] reported reasons for dropout that stemmed from dissatisfaction with the employed tool. One study [30] focused on changes in usage levels for each feature of the tool over time by assessing the number of days during the last 7 days on which each feature was used. However, the reported values were the mean and standard deviation calculated for the participants who completed each visit at 3 months and 6 months. Some participants dropped out after the 3-month visit, so the difference in reported values between two time points does not reflect usage changes for all the participants. Four studies [22,25,30,32] analyzed factors potentially associated with the level of engagement throughout the trial, but only one of them [32] also included the level of engagement for the first phase as a potential factor, which was actually the only factor that was associated with the engagement level of the later phase.

Regardless of study design, if patients are involved in a longitudinal trial of a self-management tool, mechanisms of their engagement with the tool over the period should be analyzed to identify factors associated with usage [33]: how participants used the tool; why they used, continued using, or stopped using the tool; what they experienced by using the tool; and how they perceived the tool. Many studies of Web-based health-promoting programs investigate relationships between attrition and user characteristics or design factors of the program, for example [34-39]. Especially for the early stage of development or a feasibility study, such mechanisms of engagement should be qualitatively analyzed in regard to the design elements of the tool from the perspective of user-centered design. For example, based on a 13-week field trial of DiasNet mobile by one patient, Jensen and Larsen showed that combining a usage log and subsequent interview provided good insight into usage [40]. Such qualitative analysis will not only identify design issues to be improved for better usability, but will also enable researchers to list factors to be statistically investigated for their association with engagement with the tool in a larger study. To our knowledge, no standard measure has been specifically designed to assess usability of a mobile-phone-based self-management tool for diabetes. Garcia et al [8] presented a heuristic evaluation method for mobile application for self-management of Type 1 diabetes mellitus (T1DM). Based on evidence-based guidelines and iterative discussions with patients and physicians, Chomutare et al [6] suggested a list of features that a mobile diabetes application should have. In addition to these methods, elaborating on participants’ usage and experience with a tool over time will help in accumulating knowledge that will establish a solid outcome measure for feasibility and usability. Such a knowledge base will also help researchers, clinicians, designers, and developers to choose or design a suitable tool for their purpose.

The authors of this paper have developed ICT systems to support sustainable self-management of T2DM, emphasizing unobtrusiveness in patients’ daily life and simplicity for ease of use. From a very early stage, the design process has involved patients with T2DM as prospective users. A self-management tool, the Few Touch application, was developed for continuous use with the purpose of improving users’ blood glucose management by increasing physical activity and encouraging a healthier diet. The feasibility of the application was tested by the 12 participants in their real-life settings for half a year as the final part of the design process [41,42]. Even at the completion of the initially planned half-year testing, all the participants showed strong interest in continuing use of the application and further participation with the study. However, a decreasing tendency in measurement frequency was generally observed in statistical analysis of aggregated blood glucose readings by all the participants for 1 year [43]. In the present study, therefore, we explored mechanisms of participants’ engagement with the application. We identified design factors associated with long-term usage and usability of the application by conducting a thorough analysis of results from multiple types of investigation focused on each participant’s usage, experience, and perception of the application over time. By elaborating on the results of the above-mentioned analysis, this study aims to contribute toward accumulating knowledge about factors associated with use of a mobile self-management application as well as to disseminate the value of involving patient-users from an early design phase to a longitudinal trial of the product for the very last design iterations.

## Methods

### Long-Term Trial of the Few Touch application

The Few Touch application was tested for 1 year by 12 individuals with T2DM (4 men and 8 women; age ranged from 44 to 70 with a mean age of 55.1 (SD: 9.6) and mean disease duration was 8.1 (SD 3.8) years at the beginning of the long-term trial) who had been involved in the design process. We use the term “participants” for these 12 individuals. The local regional ethical committee approved the study protocol in 2006 (Regional komité for medisinsk forskningsetikk Nord, Ref. No. 13/2006). The recruitment process and other details about the participants are explained elsewhere [41].

The main component of the Few Touch application is the smartphone-based “Diabetes Diary”. Core features of the Few Touch application are: (1) automatic wireless data transmission from a blood glucose meter and a step counter, (2) nutrition habit recording enabled by few-touch operation on the smartphone, (3) feedback with simple analysis of these three types of data shown by the Diabetes Diary, (4) goal-setting functions for step counts and nutrition habits, and (5) general tips function for self-management of diabetes.

**Figure 1:**
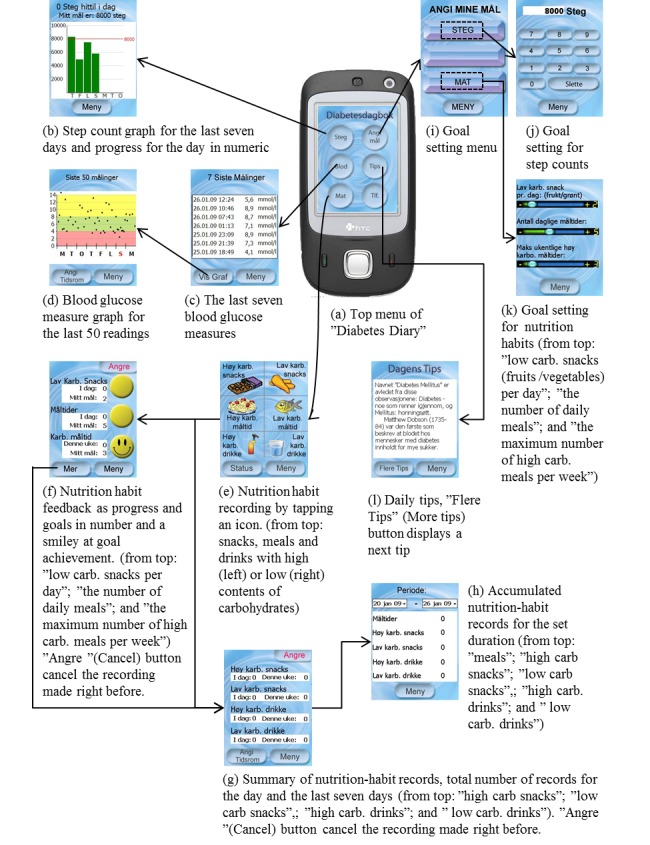
Screen design and structure of Diabetes Diary (“Diabetesdagbok”).

Figure 1 shows the structure and screenshots of each page in the Diabetes Diary. The “Phone (tlf)” button switches to the default top menu of the smartphone. Tapping the button “Angi Tidsrom” (change period) on screen (d) displays a blood glucose measure graph showing all the data for the set duration. Tapping “lav karb. (low carb.) snacks”, icons for meals, or the “status” button displays page (f). Tapping “høy karb. (high carb.) snacks” or icons for drinks displays page (g). Details of each function and the design process of the Few Touch application including reasons for the choice of technologies can be found elsewhere [41,42,44-47].

The schedule for the long-term trial and the timing of data collection is summarized in Table 1. At the introduction of the Few Touch application in September 2008, the authors explained that the frequency of using the system was up to the participants. Instead of providing detailed instructions for using the nutrition habit recording system, we challenged participants to record what they ate and/or drank in a way that was relevant for them, so that this process could evoke reflective thinking [48]. Due to the limited battery life of the step counter and the limited human resources after the end of the 6-month trial planned initially, all the step counters stopped working before the meetings held in October 2009.

**Table 1:** Schedule for the long-term trial and the timing of data collection.

Meetings	Time (month, year)	Events
1	September 2008^ a^	Introduction of the Few Touch application (except physical activity sensor system and tips function)
		Questionnaire 5
2	October 2008 (7 weeks after Meeting 1)	Introduction of tips function
		Focus group sessions (the participants were divided into two groups)
3	December 2008^b^, January 2009^c^	Introduction of physical activity sensor system
		Individual semistructured interview
		Questionnaires 4 and 7
4	March 2009	Focus group sessions (the participants were divided into two groups)
		Questionnaires 1, 2, 4-8
		System Usability Scale (SUS) [49]
5	June 2009	Focus group session^d^
6	October 2009	Focus group sessions (the participants were divided into two groups)
		Questionnaires 3-7, 9

^a^For P07 and P11, the application was introduced on October 1 and 7, 2008, respectively

^b^Two participants attended an individual meeting.

^c^Ten participants attended an individual meeting.

^d^Ten participants attended the focus group session.

### Data Collection and Analysis

#### Usage Trends and Patterns

Recorded data in the Diabetes Diary, comprising blood glucose measures and step counts that had automatically been transferred to the users’ smartphones and manually recorded nutrition habits, were collected in every meeting after each function became available. To explore usage trends over time, we defined “usage rate” as the number of days per week on which each function was used. For the physical activity system, unless participants reported any problems with it, we assumed that days with step counts greater than zero were the days on which the system was used, because the step counter automatically transmits data once a day at a regular time, even if it has not been used on that day. To evaluate usage trends (Multimedia Appendix 1), we employed the Mann-Kendall trend test [50] on usage rates for weeks in which each function was available for 7 days. This is a non-parametric test with the null hypothesis that the signs of single differences in target values sum to zero. Thus a significant result indicates an average trend in either direction. The test statistic tau is a measure of the monotonicity of the trend. Tau=1 means a monotonic increase; tau=0 indicates no trend either way; tau= -1 means a monotonic decrease. To examine overall levels of usage throughout the trial period, we looked at the number of days on which each function was used against a period in which each function was available. To investigate each participant’s daily usage pattern and its change over time, we focused on the distribution of time points during the day for blood glucose measurements and nutrition habit recordings throughout the trial. We applied the same method used in [43], a kernel density estimator with Gaussian kernel smooth, on the time at which recordings were made by each participant. Each dataset for blood glucose measurements included all the data collected during the trial duration, while we divided data for nutrition habits recording into 2-month intervals in order to highlight the change over time. To find the bandwidth that highlighted characteristics of usage patterns in the best way, we tried different bandwidths, such as 0.1, 0.5 and 1 hour, on all datasets. As a result of this process, we selected 1 hour for the bandwidth for all the calculations. For blood glucose measurements, we also looked at the daily frequency of measurements.

#### Questionnaires

Although the questionnaires that we used covered a variety of aspects, this paper focuses on reporting the results regarding the participants’ perception of usability and usefulness of the Few Touch application. Both standard and tailored questionnaires were administered. To evaluate the usability of the whole system, at Meeting 4 we administered the System Usability Scale (SUS) [49], which is widely used [51] and has been found valid [52]. Inspired by the conclusion from the case study [53], we designed and administered original questionnaires to investigate usability in more detail based on the context of the Few Touch application. The following is a list of original questionnaires that are used in this study. Questionnaires 1 and 8 comprise particular items that had been found essential or important as a mobile terminal-based self-help tool in our survey of other relevant studies [9].

1. Satisfaction with 14 design elements of the Few Touch application (5-point Likert scale)
2. Agreement with motivational effect of each function on better self-management (5-point Likert scale)
3. Agreement with effect of using the Few Touch application on behavior change in activities for self-management of diabetes (5-point Likert scale)
4. Perceived usefulness of the Few Touch application. (7-point Likert scale)
5. Satisfaction level with knowledge about diabetes and with the skills in diabetes management (5-point Likert scale)
6. Expected frequency of usage of the Few Touch application in future (multiple choice from: Daily, Weekly, Monthly, or Seldom)
7. Satisfaction level with the tips function (5-point Likert scale)
8. Agreement with possible improvement of the Few Touch application by incorporating 10 potential functionalities (5-point Likert scale)
9. Agreement with actual improvement in medication, blood glucose control, physical activity level, and nutrition habits (yes/no)

#### Interviews and Thematic Analysis of Collected Data

Semistructured interviews were conducted at Meetings 2-6. The questions used in the interviews were designed to identify how the participants used and experienced the Few Touch application in relation to self-management activities in terms of the whole application, each function, and usability of both the application and the smartphone. All the interviews were voice recorded. Because the questions were strongly connected to the aim of the analysis, we examined data from interviews by following the framework suggested by Braun and Clarke [54], in which codes and themes were identified at semantic level using a theoretical approach. The findings from the interview data were investigated by collating both the results of questionnaires and the results of usage patterns and trends. Identified themes were arranged into a structure that explained mechanisms of participants’ engagement with the application. To identify factors associated with participants’ engagement with a mobile self-management application, data extracts relevant to the application design were mainly used together with answers to relevant questionnaires.

## Results

In this section, we use the code “Pxx” to indicate a specific participant, where “xx” shows the participant’s ID number.

### Usage Trends and Patterns

Results from the Mann-Kendall trend test on usage rates confirmed a significantly decreasing (*P*<.05) usage trend among 10 out of the 12 participants, though the magnitude of the decrease varied widely (Table 2). The generally decreasing trend is in line with the result for blood glucose measurements found previously [43]. However, the analyses of datasets by participant revealed that some of the participants used functions constantly and in a very determined way during the trial period. Table 3 summarizes the numbers of days on which each function was used against a period during which each function was available. Both Table 3 and trends in usage rates (Multimedia Appendix 1) show that P03 and P09 used both the blood glucose sensor system and the physical activity sensor system very consistently.

**Table 2:** Results from Mann-Kendall trend test on usage rate.

	Blood glucose sensor system		Nutrition habit recording system		Physical activity sensor system	
Participant	Tau-value	*P* value	Tau-value	*P* value	Tau-value	*P* value
P01	-0.19	.06	-0.58	<.001	-0.57	<.001
P02	0.22	.03	-0.01	0.91	-0.10	0.46
P03	-0.01	.96	0.16	0.14	0.21	0.16
P04	-0.35	.002	-0.37	<.001	-0.62	<.001
P05	-0.41	<.001	-0.18	0.07	-0.16	0.18
P06	-0.31	.003	-0.39	<.001	-0.43	0.001
P07	-0.11	.33	-0.58	<.001	-0.58	<.001
P08	-0.06	.56	-0.34	.002	0.12	0.47
P09^a^	-0.05	.70	-0.37	.002	-0.35	0.08
P10	-0.54	<.001	-0.42	<.001	-0.35	0.01
P11	-0.45	<.001	-0.71	<.001	-0.27	0.05
P12	-0.63	<.001	-0.61	<.001	-0.07	0.69

^a^All the recorded data on P09’s smartphone were accidentally deleted at Meeting 2, and only data recorded after Meeting 2 were used for analyses.

**Table 3:** The numbers of days on which each function was used against a period in which each function was available.

	Blood glucose sensor system			Nutrition habit recording system			Physical activity sensor system		
Participant	Dr^a^	Da^b^		Dr	Da		Dr	Da	
P01^d^	102	395	(26%)	101	395	(26%)	71	152	(47%)
P02^d^	158	393	(40%)	51	393	(13%)	56	239	(23%)
P03^d^	390	395	(99%)	365	395	(92%)	210	219	(96%)
P04	16	393	(4%)	11	393	(3%)	33	138	(24%)
P05^d^	294	393	(75%)	277	393	(70%)	143	265	(54%)
P06	334	393	(85%)	323	393	(82%)	159	244	(65%)
P07^d^	327	374	(87%)	98	374	(26%)	61	202	(30%)
P08^d^	58	395	(15%)	357	395	(90%)	161	197	(82%)
P09^c, d^	348	352	(99%)	8	352	(2%)	129	132	(98%)
P10^d, e^	278	389	(71%)	88	393	(22%)	116	191	(61%)
P11^d^	60	380	(16%)	152	380	(40%)	86	210	(41%)
P12	209	393	(53%)	240	393	(61%)	86	147	(59%)

^a^Dr is the number of days on which records were made.

^b^Da is the number of days when a function was available.

^c^All the recorded data on P09’s smartphone were accidentally deleted at Meeting 2, and only data recorded after Meeting 2 were used for analyses.

^d^The step counters had problems, so that there were periods when participants could not use their step counter.

^e^P10’s blood glucose sensor system did not function for 4 days.

**Figure 2:**
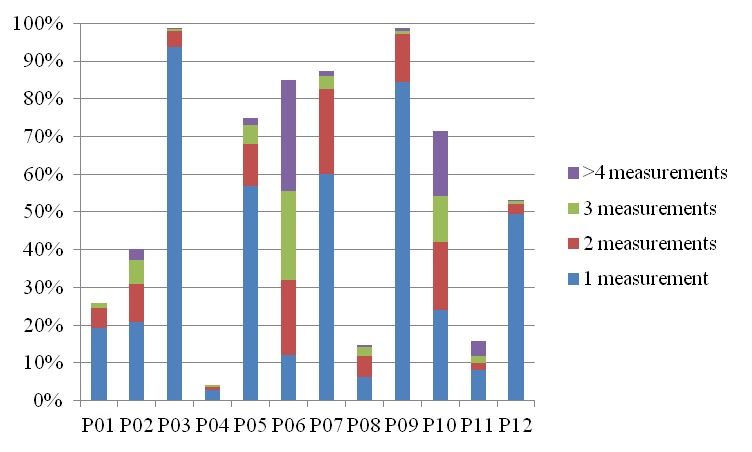
Distribution of blood glucose measurement frequency among days on which any blood glucose measurement was performed.

Figure 2 shows the distribution of blood-glucose measurement frequency among days on which any blood glucose measurement was performed. From Figure 2, it is clear that P03 and P09 measured once a day for most of the days on which they had measurements (370 out of 390 days (95%) for P03 and 297 out of 348 days (85%) for P09). On kernel density estimates of distributions of time points at which blood glucose was measured (Multimedia Appendix 2), a significant single peak can be identified in the morning time for P03 and P09, which demonstrates that they are habituated to measure blood glucose in the morning almost every day. Including P03 and P09, a significant peak in the morning time was observed among most of the participants, which is also in line with the result from aggregated data for all the participants [43]. However, the kernel density estimates (Multimedia Appendix 2) also revealed that the patterns of times at which measurements were taken were quite different from one participant to another, which reflects different needs for blood glucose measurements. For example, P06 and P10 used the blood glucose sensor system for more than 70% of days when the function was available, and often they measured blood glucose more than once a day (Figure 2). From the kernel density estimates (Multimedia Appendix 2), it is observed that P10 measured in the morning more or less regularly and otherwise rather sporadically, while P06 usually measured very sporadically. P01 used the function moderately: once a day for most of the times when used (75%, 76 out of 102 days) (Figure 2). The two sharp peaks for P01’s density estimates (Multimedia Appendix 2) indicate that P01 often measured either in the morning or in the evening.

Regarding nutrition habit recordings, we could observe a change in usage patterns for some participants (eg, P01, P07, and P11) in a relatively early phase (Multimedia Appendix 3). This corresponds to what 4 participants (P01, P05, P06, and P11) told us at Meetings 2 and 3: they tried to record the data right after eating, but sometimes they recorded it at the end of the day to summarize their food intake, while P07 shifted recording activity to morning time. After updating the user interface of nutrition habit recording at Meeting 4, which is explained in detail in a later subsection, P08 clearly changed his/her way of nutrition-habit recordings from “right after every eating/drinking” occurrence to “summarizing at the end of the day”, whereas this modification did not seem to influence either usage patterns or usage rate for the other participants.

Regarding the physical activity sensor system, 9 (P01, P02, P03, P05, P07, P08, P09, P10, and P11) of the 12 participants had problems and their step counters were repaired or replaced. The major problem was battery attrition of the step counter. A significant decreasing usage trend (*P*<.05) was observed among 5 of the participants, which is less than for the other two functions. However, this result may need to be interpreted carefully due to the much shorter period in which the physical activity sensor system was available than the other two functions.

### Questionnaires

The Few Touch application was generally perceived as satisfactory. The perceived usefulness of the whole application by the participants remained considerably high over time (Table 4), though several of the participants expected the frequency of usage to decrease over time (Multimedia Appendix 4, Questionnaire 6). The average score for the SUS questionnaire was 84.0 (SD: 13.5, range: 67.5-100) [41,42]. This score is high compared with other studies where the SUS questionnaire was used, considering the average score together with the conclusion on usability in each study [55-57]. The result from Questionnaire 1 shows that only one user expressed dissatisfaction about the size of the mobile phone, which is 107 x 55 x 16 mm and weighs 120 gram. No other items were perceived as explicitly unsatisfactory for the Few Touch application in this questionnaire.

**Table 4:** Questionnaire 4—Distribution of the answers to questionnaire about perceived usefulness of the Few Touch application (1: Not useful at all, 7: Very useful).

Elapsed time / Usefulness of the application	1-3	4	5	6	7	Mean
3-4 months (Meeting 3)	0	1	0	6	5	6.3
6 months (Meeting 4)	0	1	1	2	8	6.4
1 year (Meeting 6)	0	0	1	4	7	6.5

The blood glucose sensor system was perceived as the most motivating, followed by the physical activity sensor system and the nutrition habit recording system (Multimedia Appendix 4, Questionnaire 2). In contrast, the participants expressed lukewarm perceptions regarding the effect of using the Few Touch application on achieving adequate frequency of blood glucose measurement (Multimedia Appendix 4, Questionnaire 3). Likewise, only half of the participants answered that their blood glucose control had been improved, while slightly more participants answered that their physical activity level and nutrition habits had been improved in the course of 1 year (Multimedia Appendix 4, Questionnaire 9). No participants answered that their medication had been improved, but P03 (at Meeting 3) and P11 (at Meeting 4) mentioned in an interview and a focus group, respectively, that the number of tablets had been reduced. Also, overall satisfaction levels with their knowledge about diabetes and with skills in diabetes management were not drastically changed in the course of 1 year (Multimedia Appendix 4, Questionnaire 5). This is also reflected by the fact that participants’ satisfaction level with the tips function decreased over time (Multimedia Appendix 4, Questionnaire 7). Although the participants considered the tips concise and useful, 5 participants (P01, P03, P05, P09, and P12) suggested improvements, as reported previously [42].

The results of the questionnaire that addressed the participants’ preferences for potential functionalities of the Few Touch application are shown in Multimedia Appendix 4, Questionnaire 8, ranked by the total score. “A smaller step counter that is easier to wear” was highest rated, and “automatically popping-up tips” ranked as second. The other items that no one disagreed with were “automatic” functions. Besides “use of own mobile phone”, all items that were disliked by some of the participants were functions that involved communication with other people. Most of the participants stated that using the application as a “self-help” tool was enough for them, though collaborative use of the application might be motivating.

We could not observe any deterministic associations between answers to any questionnaires and usage of functions.

### Interviews and Thematic Analysis of Collected Data

#### Mechanism of Engagement With the Few Touch application

**Figure 3:**
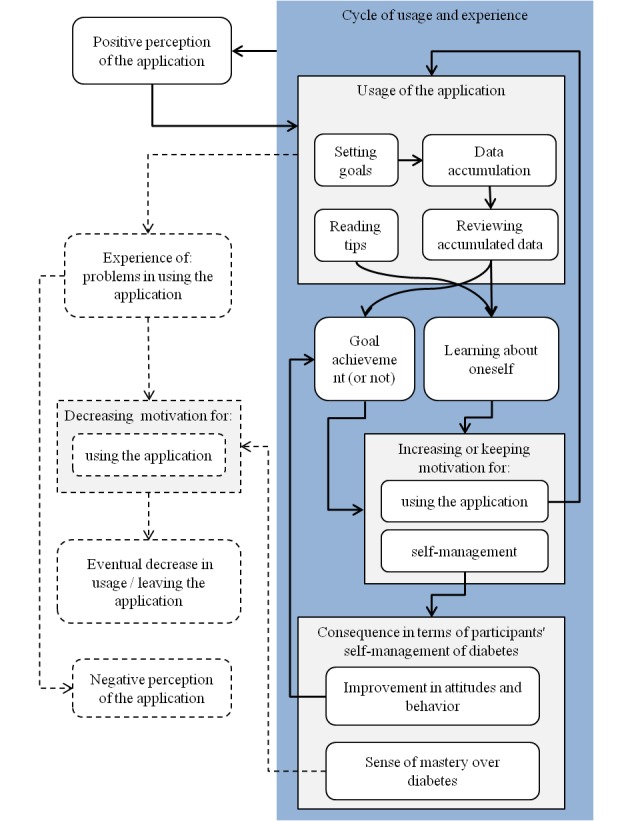
Mechanism of participants’ long-term engagement with the Few Touch application.

The mechanism of participants’ long-term engagement with the Few Touch application is illustrated in Figure 3. Note that Figure 3 was drawn to explain how participants used the application, what they experienced as a result of using the application (such as behavior change), and how they perceived the application. For this reason, each block or group of blocks in Figure 3 does not necessarily correspond to an identified theme in the thematic analysis. Multimedia Appendix 5 summarizes prominent themes, codes, and examples of quotes.

First, we could identify a cycle of usage of the application, experience, and impact of using the application expressed as elements in a box with a dark background in Figure 3. The Few Touch application was developed with the purpose of motivating people to use the tool so that they would benefit by using it over a long time. The thematic analysis indicated that the Few Touch application played a role as a flexible learning tool by which individuals with T2DM could instantly confirm how their self-management activities and other health conditions such as illness influenced their blood glucose levels. Despite a rather high age for some of the participants, all the participants were able to use the chosen smartphone and the application. Patterns of engagement with the application varied widely depending on their needs: Intensive use to find relationships between their self-management and blood glucose levels; constant and regular use for gaining an overview of blood glucose values and self-management activities over time; sporadic use in out-of-the-ordinary situations such as dining out or travel with others that limited participants’ opportunities for self-management activities; and use for experimental purposes. Such a wide variety of usage shows that the application was used flexibly according to users’ spontaneous purposes as well as for regular self-monitoring every day. Depending on their status, the participants increased their motivation for either maintaining their good habits or improving their attitudes and behavior, and eventually experienced a sense of control over their diabetes. This cycle caused positive perceptions of the application, as shown in results of questionnaires. The positive perceptions contributed to further use of the application to a certain degree. This cycle of positive flow expressed by bold arrows in Figure 3 explains the mechanism of participants’ long-term engagement with the application.

Figure 3 also describes the process in which participants’ usage of the application decreased over time. Elements with dashed lines show the process of decreasing usage. Two major reasons for the decrease in usage were identified: The first was loss of motivation to continue using the application after gaining a sense of mastery over diabetes. Some of the participants interpreted “learning about oneself” with the application as “short-term use of the application until one gets control over one’s diabetes”. The second reason was problems in using the application, which is explained in detail in the next subsection.

#### Factors Associated With Usability and/or Usage Over Time

Despite generally high satisfaction with the application, experiences of problems were also reported. Problems that stemmed from outside the design concepts included battery attrition for a step counter and a Bluetooth adapter, some features of the provided smartphone that had taken some time to get used to for the first month, and problems with the smartphone. Other problems stemmed from a mismatch between design concepts and reality (Table 5). These problems caused difficulty in integrating use of the application into everyday life. Not all the issues in Table 5 were clearly specified as direct reasons for the immediate decrease in usage, but they at least degraded the usability of the application. For example, P08 admitted at Meeting 6 using the step counter extensively, despite having problems in attaching the step counter, and used it in either a pocket or a handbag. Usage attrition for the physical activity sensor system for P06 is clear compared with P08 (Table 2, Multimedia Appendix 1). Some issues in Table 5 influenced long-term usage in terms of attrition of enthusiasm. For example, at Meeting 4, P01 expressed a positive perception of the nutrition habit recording system as “it has worked” despite forgetting to record data and complaints about the categorization. However, at Meeting 5, P01 told us “it does not work for me, at least” because “it is impossible to record for the past dates though it is easy to forget recording”. This was also seen in P01’s usage rate for the nutrition habit recording system, which was zero for most of the weeks since week 37.

From data extracts relevant to the application design and results from questionnaires, we identified the following factors associated with usability and/or usage over time: (1) integration with everyday life, (2) automation, (3) balance between accuracy and meaningfulness of data with manual entry, (4) intuitive and informative feedback, and (5) rich learning materials, especially about foods.

**Table 5:** Functions and features that caused deteriorated usability of the Few Touch application.

Function and feature	Design concept	Reality	Affected components in usability
User interaction design enabling nutrition habit recording completed by just one press on the appropriate category.	Users would record each meal, snack and drink immediately. Users could record food or drink intake with minimum effort.	Participants made several records at a time or recorded nutrition habits at the end of the day to summarize their food intake so that they needed more operations at a time. (P01, P03, P05, P06, P08, P10 and P12, Meeting 2) It was not always possible to record right after eating or drinking, or due to constraints of time and place. (P07, Meeting 6)	Efficiency, flexibility
Categorization of nutrition habit recording	Categories would correspond to types of eating habits that should be improved in context of T2DM, so that it encourages users to have a healthier diet.	The categorization was not precise enough for their reflective thinking, or it did not match the participants’ individual preferences based on their accumulated personal experiences. (P01, P02, P08, P11 and P012, Meeting 4)	Effectiveness, flexibility
Step counter attached on belt	A physical activity sensor should be integrated with their daily tools and outfits.	One participant (P06) did not use a belt normally. P06 had used it in a bag, but it was easy for P06 to forget about using the step counter on the next day. (Meeting 6)	Satisfaction
Step counter as a physical activity sensor	Physical activity sensor system should provide easily interpretable values to motivate a user to monitor.	The fact that other types of sports (skiing) or physical activities were not measured was disappointing. (P11, Meeting 4; [41,42] P12, Meeting 6)	Effectiveness, satisfaction
User interface of tips function and its contents	Tips function would provide a user with concise information that can be shown on a screen without necessity of scrolling or more manual operation than one button press to access to a “tip of the day”.	Participants wanted better access to information that they want to read (P05, P08, and P09, Meeting 5)	Efficiency, satisfaction
		Participants wanted more and richer information (P01, P03, P09, and P12, Meeting 4), preferably delivered by SMS with tailored contents based on user’s profile (P12 [42])	Satisfaction
Diabetes Diary as a software on a smartphone	Users would easily access to their records and information relevant to self-management of diabetes by integrating necessary functionalities into a software application running on their personal mobile phone.	A participant (P04) stopped using the smartphone as his/her personal mobile phone, because it had problems as a mobile phone (Meeting 6)	Effectiveness, efficiency, satisfaction

##### Integration With Everyday Life

The participants generally appreciated the minimal effort required for keeping track of self-management activities and for referring to them, which is the design concept achieved in the user-involved design process. Instant access to the application on the smartphone that was used as a personal mobile phone played a great role in integrating the application use into everyday life. This is supported by the fact that no participants used the history view function on the blood glucose meter and by the fact that P04, who had problems with the provided smartphone, did not continue using the application.

**Figure 4:**
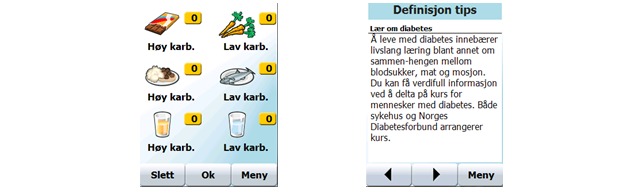
Modified user interface for nutrition habit recording (left), and the tips function (right).

Many of the issues listed in Table 5 also illustrate the importance and difficulty of application design that can be easily integrated into each individual’s everyday life. At Meetings 4 and 5, we updated the user interface design of some pages due to problems that were reported. A page for nutrition habit recording was originally designed to enable recording with minimum effort. However, this design actually made nutrition recordings more cumbersome when a user wanted to record more than one nutrition habit as a summary of a day. The update (Figure 4, left) at Meeting 4 enabled entry of more than one eating or drinking record at a time. Users could press each button the appropriate number of times and then press the “OK” button to record the data. The number of times that each button had been pressed was displayed in the yellow box next to the button. To reset the number of times to zero, users could press the “Cancel” (“Slett”) button. Navigation in the tips function was also updated at Meeting 5 because the tips function was used for look-up despite being designed to give a “daily” tip. We added a “Back” button as well as a header and category name to each tip to make it easier to view the content and find information (Figure 4, right).

##### Automation

Automation of data transfer from the blood glucose meter and the step counter played a key role in making the use of the application as effortless as possible. The participants appreciated not only that they did not have to write down the values any more but also the fact that the graphical feedback was automatically prepared based on the transferred data. Results from Questionnaire 8 also support the importance of automation.

An interesting change over time in perceptions of usefulness was observed regarding automation of recording and data visualization. P10 told us in the Meeting 2 that s/he had used to write a very precise paper diary before the trial started, so s/he found no difference in the Diabetes Diary, and even the blood glucose graph did not provide anything new. However, at Meetings 3 and 4, P10 told us of now, unlike earlier, appreciating the blood glucose graph to see how his/her blood glucose varied and to relate it to food consumed. Finally, at Meeting 5, P10 told us that s/he had recently stopped writing down measurements manually, which s/he had continued just as a habit, due to now relying on the application. P03 admitted to continuing to write down blood glucose values for a while, but s/he became used to getting the values on the smartphone.

##### Balance Between Accuracy and Meaningfulness of Data with Manual Entry

Although most of the participants liked and wanted to keep the simplicity of the system for nutrition habit recording, some of them found that the categorization for this system was not appropriate for their reflective thinking or that it did not match their individual preferences based on their accumulated personal experiences (Table 5). The thematic analysis showed that this became more evident and crucial in terms of usage in the latter half phase of the trial compared with the early phase. Participants indicated their need for a function enabling more detailed recording or a function to customize food types on the nutrition habit recording system without jeopardizing the simplicity of the system. Such needs correspond to the wide variety of usage of the application described earlier.

##### Intuitive and Informative Feedback

The participants showed different preferences for the design of feedback depending on function. At one of the focus group sessions in Meeting 4, all the participants stated that feedback showing progress toward goals was most important for encouraging daily physical activity and good nutrition habits. They also mentioned that they rarely used screen (h) in Figure 1 where they could refer to accumulated data for a period that they had set. Only 1 participant expressed the need to view the history of step counts older than a week. In contrast, the distribution of historical blood glucose measures on the background divided into three colors was perceived as intuitive and informative, enabling users to determine whether they were “doing all right” over time.

Though some other participants stated that the system was simple enough for them to see that their self-management activities influenced their blood glucose levels, 2 participants (P05 and P11) clearly expressed their need for improvement of the feedback design so that it visually showed the relationship between the three components: blood glucose level, physical activity, and nutrition habits. Both participants mentioned their difficulty in maintaining their focus and motivation in continuing self-management activities. At Meeting 3, P05 also mentioned the importance of keeping a self-management tool simple so that s/he would not become confused with complicated information. One of the suggestions for improvement of the application included a function to show a filtered list of fasting blood glucose measurements only, which also illustrates a need for feedback to be more informative.

##### Rich Learning Materials, Especially about Foods

Most of the participants appreciated the tips about food, and even more enriched content was requested. Many of the suggestions for improvement of the application concerned functions or learning materials about foods.

## Discussion

### Main Findings

Together with qualitative inquiries, detailed quantitative analyses on each participant’s usage of the provided tool gave us insights into mechanisms of participants’ engagement with the tool and led us to a better understanding of factors associated with usage and usability over time.

The Few Touch application served as a flexible learning tool for the participants depending on their spontaneous needs as well as for regular self-monitoring. Usage of the application was supported by the minimum effort required for keeping track of self-management activities and for referring to them, which was the design concept achieved in the user-involved design process. Except for a few participants, a decrease in the usage trend was generally observed. Having gained a sense of mastery over diabetes and experiences of problems were identified as reasons for decreased motivation to continue using the application. Some of the problems stemmed from a mismatch between design concepts and reality, even though the design concepts were obtained in a process involving the participants. The impact of such mismatches on usage and usability became critical over time among some participants.

In the following sections, we will discuss our findings by comparing them with relevant studies.

### Mechanism of Engagement With the Few Touch application

The learning process based on personal experiences on top of necessary knowledge provided by diabetes education builds a foundation for designing the patient’s own self-management plan. It is also claimed in previous studies that a supporting tool for people with diabetes should facilitate this learning process [48,58]. The application was perceived as easy and simple. Such characteristics played an important role not only in enhancing motivation to use the application but also in the learning process because “keeping track of performed actions in self-management activities is notoriously difficult” [48].

A wide variety of patterns of engagement with the application observed in this study is in line with findings in the two deployment studies of a health monitoring application, MAHI (Mobile Access to Health Information). MAHI was originally designed with the focus on development of reflective thinking skills through social interaction for newly diagnosed individuals [59]. The first deployment study [27] for newly diagnosed patients revealed that many participants considered that intensive and focused exchange with the educator by using MAHI would be “most beneficial at the beginning of their engagement with diabetes management”. However, their second deployment study [60] confirmed that MAHI was also well accepted by people with more extensive diabetes experience as a tool for identity construction through storytelling. Consistent with the findings from the MAHI deployments, our study support the idea that design that allows divergent interpretation supports flexible appropriation [61]

Decrease in usage after gaining a sense of mastery over diabetes is in line with findings by relevant studies [30,32,62]. The application was equipped with neither “push” factors nor an advanced decision support system providing feedback based on accumulated data and other personal profiles, which are advocated for long-term usage, according to relevant studies [62-65]. Nevertheless, usage by many of the participants remained relatively high over the 1-year trial period (see Multimedia Appendix 1). This might have been caused by intrinsically high motivation of participants in the design process. As shown in Multimedia Appendix 4 Questionnaire 8, the participants expressed preferences for such features, suggesting the role these would play for stronger engagement with the application.

### Factors Associated With Usability and/or Usage Over Time

#### Integration With Everyday Life

The mobility and pervasiveness of the smartphone as a personal mobile phone played an important role in integration of the application into everyday life. The finding strengthens the conclusions in recent studies [66,67] that smartphones would be more suitable platforms for a support tool for self-management of lifestyle-related diseases than contemporary mobile phones or PDAs.

#### Automation

In the present study, automation was successfully employed only to reduce unnecessary burden in tracking self-management activities, such as transcribing data, so that it would support longitudinal use of the application as advocated by Mulvaney et al [14] as well.

#### Balance Between Accuracy and Meaningfulness of Data with Manual Entry

Accuracy of data obtained by a sensor is critical in terms of giving proper credit to users [48,68]. On the other hand, regarding data obtained by manual entry, its accuracy level should be determined in the light of how meaningful it would be for a user who invests additional effort. The ability to customize a feature is important for the function to be meaningful for individual users, and this idea resonates with design implications described in a study by Chen et al [58].

#### Intuitive and Informative Feedback

Findings by Kelders et al [69] correspond to our finding that the participants regarded feedback showing progress toward goals as most important for encouraging daily physical activity and good nutrition habits. The perceived usefulness of visualizing trends in blood glucose levels is also in line with the finding in a study by Forjuoh et al [30]. To better support “learning processes”, visual feedback showing the historical distribution of all three factors—nutrition habits, physical activity, and blood glucose level—would have made it easier to find relationships between them, especially for the participants who had difficulty in keeping focus or tended to easily lose motivation. This implication is consistent with findings by Russell-Minda et al [15] regarding the importance of usability to patients who need encouragement or help in self-management activities. Designing visually integrated feedback for all three factors incorporating a time perspective would however be a great challenge. In addition, such design needs to be carefully developed to avoid the risk of inadvertent reinforcement of “individuals’ preconceived notions and biases” that lead to a wrong assumption between their self-management activities and blood glucose level [48].

#### Rich Learning Materials, Especially About Foods

Findings by Kanstrup et al [70] also show clear needs by participants for “access to information about particular things of importance, eg, the ingredients in food to make more qualified decisions”. This implies that the participants experienced a need to learn more about food by referring to external information in their reflective thinking process. This is consistent with findings by Savoca et al [71] that not only the patient’s experimentally accumulated personal knowledge about the relationships between foods and health, but also external knowledge of a recommended diet, comprise a part of complex and dynamic processes of behavior change in diet. Given that behavior change in diet is the most challenging of the self-management activities and that lack of knowledge about diet is the highest ranked barrier [72], this implication is plausible.

### Lessons Learned: Long-Term Engagement of Patient-Users in the Designing Process

This study confirmed the importance of involving “patient-users”, not only in the specification-design phase but also in the trial phase of a working prototype in a real-life setting for enough time to clarify how the chosen design works in relation to expectations and how the design can be improved [40]. Mismatches between design concepts and reality can happen even though the same participants are involved in both design-concept making and a trial. Their impact on usability and usage may become critical over time. When technical or financial constraints hinder realization of obtained design concepts through patient-user involved processes, such as our case of step counters and choice of a smartphone model, it is especially important to examine how great the impact of mismatches would be on both usability and usage in the long term. Therefore, if possible, it is wise to continue involving the same patient-users so that effective feedback will be gained during the final cycle of design iterations.

To achieve such involvement of patient-users requires their strong and long engagement in the process. The strong engagement of the participants in this study was achieved through a variety of efforts by the researchers [42]. Among them, offering frequent opportunities to meet played an important role in motivating the participants to stay in the study, as it is one of the factors influencing nonusage attrition and dropout attrition in eHealth trials [33]. Frequent meetings also facilitated quick responses to the participants’ feedback by improving the prototype and by organizing new inquiries for further iterative design processes. The participants could feel that they were really contributing to the designing process through these interactive processes. Another advantage is that frequent meetings with other participants offer opportunities for them to learn from others. This was mentioned by some of the participants. Fudge et al [73] also found that learning from others in similar situations was one of patients’ motives to attend meetings in a user-involved program for improvement of health services for people with stroke. They discuss this issue as a concern that the ability of user involvement to improve service may be questionable, “if this (to improve service) is not the primary motivation of those involved”. In our study, we did not specifically investigate whether “learning from others” was the primary motivation for the participants to become involved in the design process or to attend the meetings. However, as far as we observed the participants in the meetings, this was a secondary effect in enhancing motivation to continue using the system.

### Limitations

Though the first author of this paper understands the Norwegian language, she is neither a Norwegian citizen nor a native Norwegian speaker. On the other hand, the second author is a native Norwegian, has T1DM himself, and has the same cultural background as the participants. In addition, the second author initiated the whole design process of the Few Touch application, from the recruitment of the participants to the development and testing of the application. The first author needed some help from the second author to interpret and understand better exactly what the participants meant in interviews. This might have caused a certain bias in the analysis.

### Conclusions

The present study showed the importance of the following two factors: (1) a thorough analysis of results from multiple types of investigations focusing on each participant’s engagement with the tool over time, and (2) involving patient-users from an early phase of design-concept making to a longitudinal trial of the system. The value of these factors was shown by the ability to identify factors that influence usability and usage in real-life settings in a long-term perspective in relation to original design concepts.

The extent to which our findings can be generalized is limited by intrinsically high motivation among many of our participants as well as the small number of the participants. However, it is highly probable that the factors that this study identified as reducing usability or usage would do so when users were less motivated. A revised version of the Few Touch application was tested by a group of 11 patients with T2DM who were not involved in the design process in order to assess the validity of our findings, and it is now being used as an intervention tool in an ongoing randomized controlled trial [74]. In that trial, a quantitative analysis needs to be carried out to investigate association between perception of features of the Few Touch application, level of engagement with the application, and the primary and secondary outcomes. Although the participants in this study are no longer engaged in design process, the Few Touch application is evolving through many research projects in which new functions are implemented and feedback for improvement is given [75]. Such series of studies will also provide useful insights into factors associated with usage and usability of a mobile self-management system.

## Multimedia Appendix 1

Long-term usage rates of each function by the participants.

## Multimedia Appendix 2

Kernel density estimates on distribution of time points at which blood glucose measurement occurred during the day along the trial duration.

## Multimedia Appendix 3

Kernel density estimates on distribution of time points at which nutrition habit recordings occurred during the day along the trial duration.

## Multimedia Appendix 4

Summary of answers to original questionnaires.

## Multimedia Appendix 5

Summary of prominent themes, codes, and examples of quotes.

## Abbreviations

ICT: information and communications technology
MAHI: mobile access to health information
PDA: personal digital assistant
SUS: System Usability Scale
T2DM: Type 2 diabetes mellitus
T1DM: Type 1 diabetes mellitus
