# Supplementary material for: Long-Term Engagement With a Mobile Self-Management System for People With Type 2 Diabetes
Source: JMIR Mhealth Uhealth. 2013 Mar 27;1(1):e1. doi: 10.2196/mhealth.2432 (PMC4114413; doi:10.2196/mhealth.2432)

Multimedia Appendix 1 – Long-term usage rates of the blood glucose sensor system (○, straight line in blue) , the nutrition habit recording system (□, dotted line in red), and the physical activity sensor system (△, dashed line in green) by the participants.

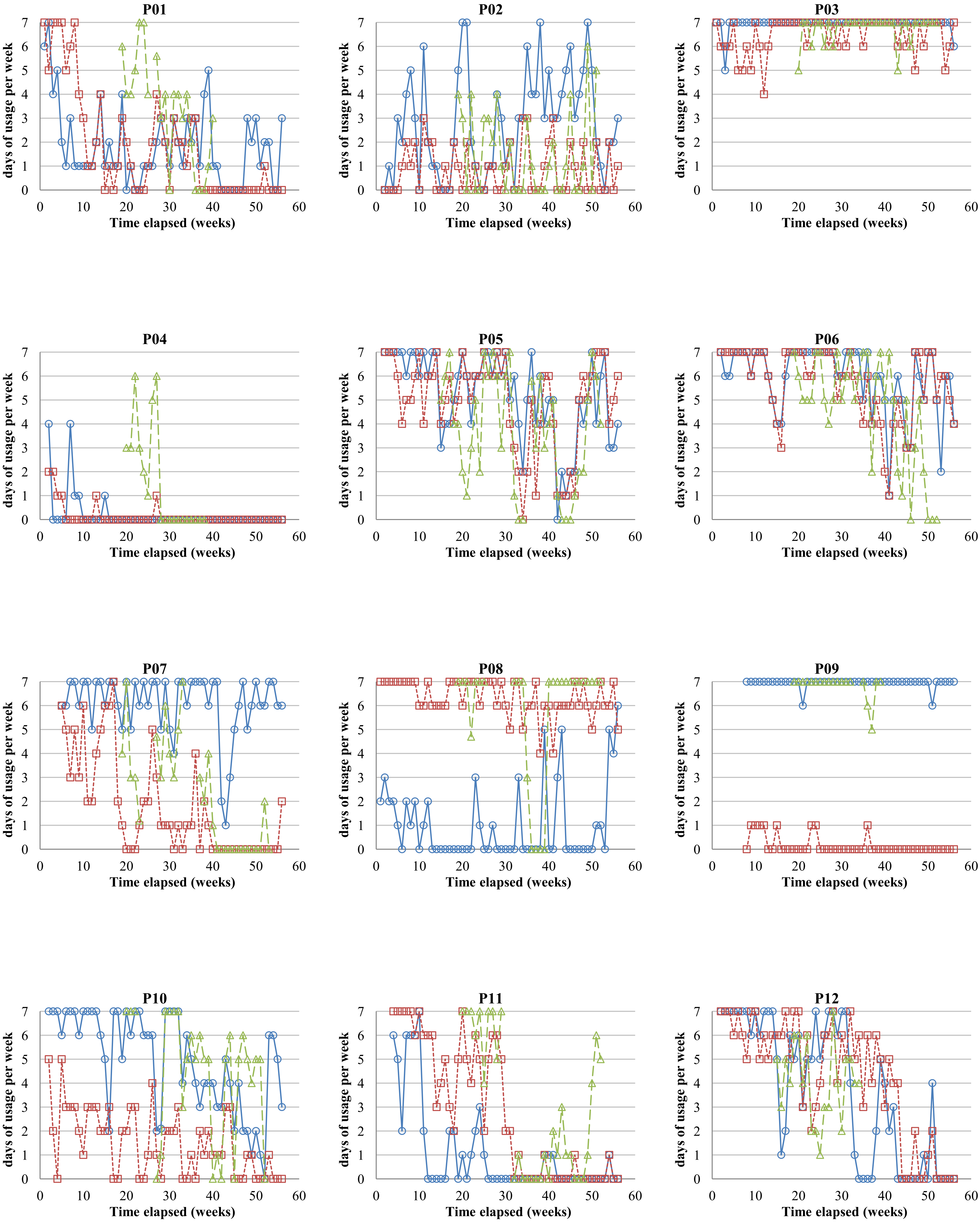

Supplement: Supplementary file 1 [file mhealth_v1i1e1_app1.pdf]
