# Supplementary material for: Long-Term Engagement With a Mobile Self-Management System for People With Type 2 Diabetes
Source: JMIR Mhealth Uhealth. 2013 Mar 27;1(1):e1. doi: 10.2196/mhealth.2432 (PMC4114413; doi:10.2196/mhealth.2432)

Multimedia Appendix 2 – Kernel density estimates on distribution of time points at which blood glucose measurement occurred during the day along the trial duration. N is the total number of data.

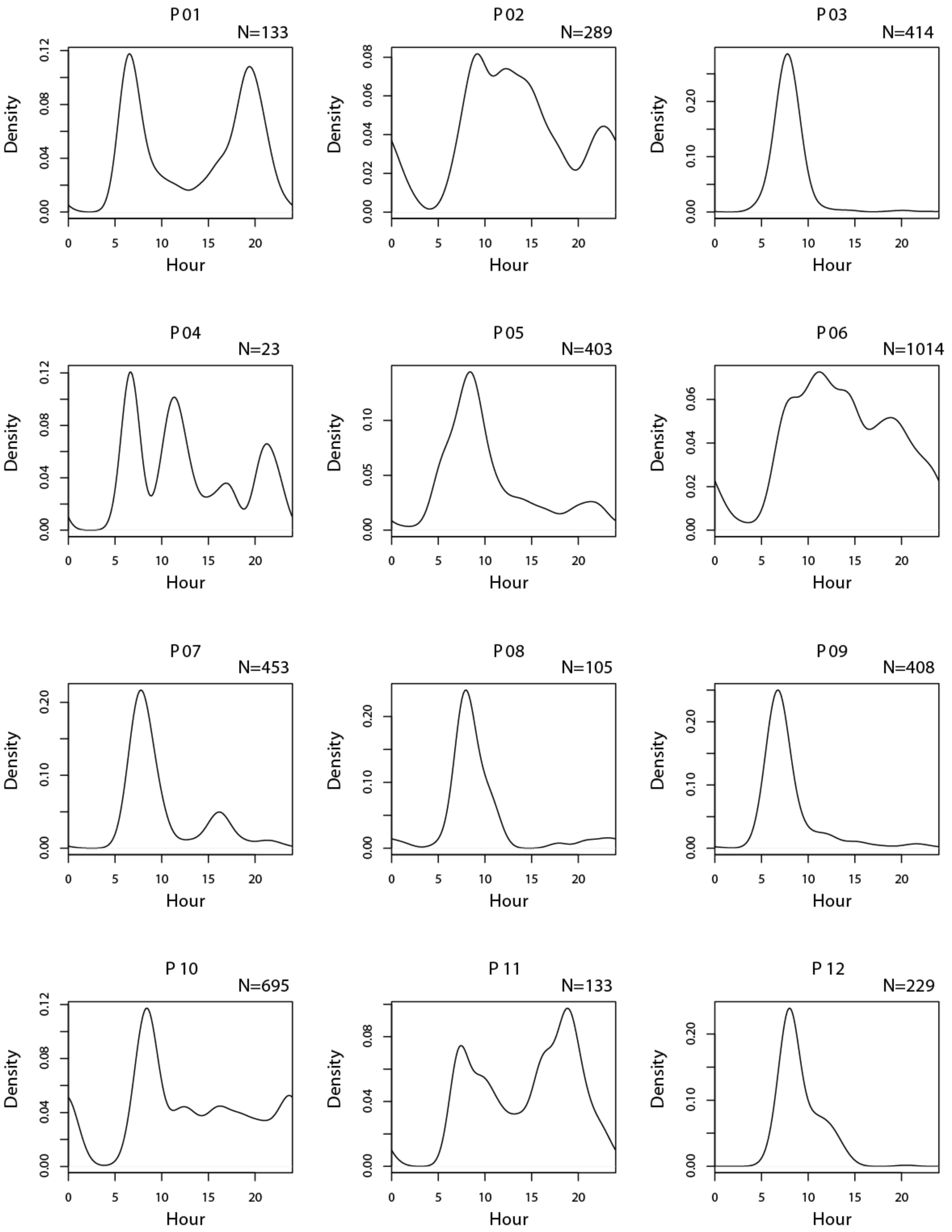

Supplement: Supplementary file 2 [file mhealth_v1i1e1_app2.pdf]
