# Supplementary material for: Long-Term Engagement With a Mobile Self-Management System for People With Type 2 Diabetes
Source: JMIR Mhealth Uhealth. 2013 Mar 27;1(1):e1. doi: 10.2196/mhealth.2432 (PMC4114413; doi:10.2196/mhealth.2432)
Supplement: Supplementary file 3 [file mhealth_v1i1e1_app3.pdf]

Multimedia Appendix 3 – Kernel density estimates on distribution of time points at which nutrition habit recordings occurred during the day along the trial duration. The legend title “mx-y” means the data from the x-th month to the y-th month. Each density has been adjusted by multiplying it by the proportion of the data amount recorded in the corresponding period in relation to the whole trial period.

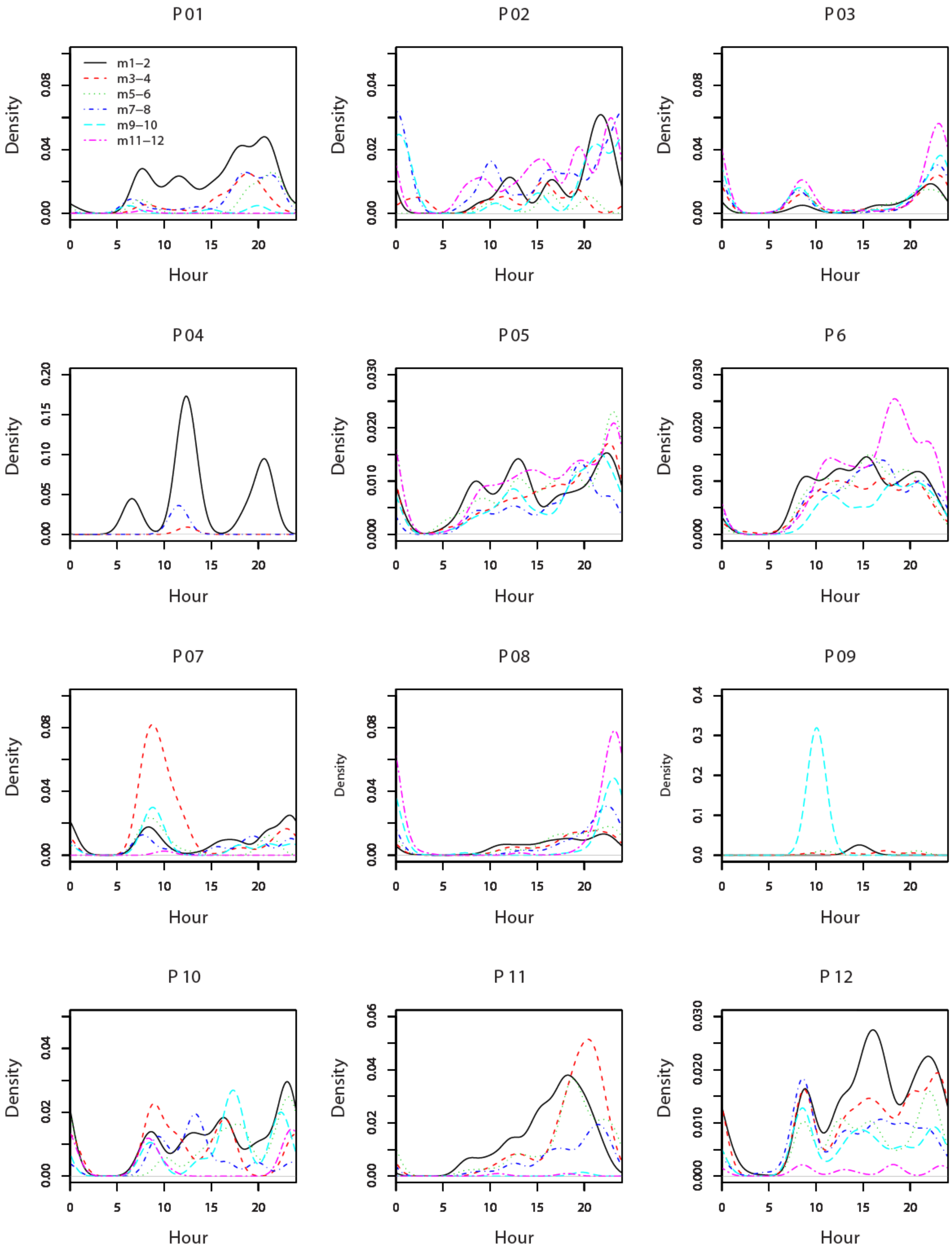

Number of recordings (N) in each period and proportion of data amount

Period	m1-2		m3-4		m5-6		m7-8		m9-10		m11-12	
Participant	N		N		N		N		N		N	
P01	316	48.5%	96	14.7%	91	14.0%	126	19.3%	20	3.1%	3	0.5%
P02	68	18.2%	33	8.8%	15	4.0%	98	26.3%	63	16.9%	96	25.7%
P03	522	10.9%	682	14.3%	646	13.5%	771	16.1%	924	19.3%	1240	25.9%
P04	39	88.6%	1	2.3%	0	0.0%	4	9.1%	0	0.0%	0	0.0%
P05	351	18.1%	312	16.0%	384	19.8%	210	10.8%	267	13.7%	420	21.6%
P06	558	18.4%	425	14.0%	502	16.5%	481	15.8%	331	10.9%	741	24.4%
P07	153	20.3%	309	41.1%	91	12.1%	91	12.1%	103	13.7%	5	0.7%
P08	668	13.4%	663	13.3%	754	15.1%	804	16.1%	796	16.0%	1298	26.0%
P09	5	7.0%	5	7.0%	4	5.6%	0	0.0%	57	80.3%	0	0.0%
P10	141	25.6%	103	18.7%	100	18.1%	68	12.3%	98	17.8%	41	7.4%
P11	257	31.7%	243	30.0%	195	24.1%	108	13.3%	3	0.4%	4	0.5%
P12	553	30.5%	416	22.9%	280	15.4%	304	16.8%	225	12.4%	36	2.0%

m1-2 (September 16 2008 – November 15 2008)
m3-4 (November 16 2008 – January 15 2009)
m5-6 (January 16 2009 – March 15 2009)
m7-8 (March 16 2009 – May15 2009)
m9-10 (May 16 2009 – July 16 2009)
m11-12 (July 16 2009 –)
