# Supplementary material for: Long-Term Engagement With a Mobile Self-Management System for People With Type 2 Diabetes
Source: JMIR Mhealth Uhealth. 2013 Mar 27;1(1):e1. doi: 10.2196/mhealth.2432 (PMC4114413; doi:10.2196/mhealth.2432)
Supplement: Supplementary file 4 [file mhealth_v1i1e1_app4.pdf]

Multimedia Appendix 4 – Summary of answers to original questionnaires

Questionnaire 1 - Distribution of the answers to questionnaire about satisfaction with elements of the Few Touch application. (1: Very dissatisfied, 2: Dissatisfied, 3: Neither satisfied nor dissatisfied / I don't know, 4: Satisfied, 5: Very satisfied) (adapted from [28, 29])

Elements / Satisfaction level	1	2	3	4	5	Mean
Color use in Diabetes Diary	0	0	0	3	9	4.8
Simplicity of navigation in Diabetes Diary	0	0	0	3	9	4.8
Text legibility	0	0	0	4	8	4.7
Ease of interpreting blood glucose graph	0	0	1	2	9	4.7
Size of display	0	0	1	3	8	4.6
Button size of Diabetes Diary	0	0	2	1	9	4.6
Ease of interpreting step graph	0	0	1	3	8	4.6
Simplicity of changing goals	0	0	1	6	5	4.3
Reaction time of Diabetes Diary	0	0	2	5	5	4.3
Size of the Smartphone	0	1	0	7	4	4.2
Simplicity of nutrition habit recording	0	0	3	4	5	4.2
Battery life of the Smartphone	0	0	3	5	4	4.2
Ease of interpreting feedback on nutrition habit	0	0	4	3	5	4.1
Step counter	0	0	4	3	5	4.1

Questionnaire 2 - Agreement with motivational effect of each function on better self-management (1: Totally disagree, 2: Disagree, 3: Neither / I don't know, 4: Agree, 5: Totally agree)

Functions / Agreement level on motivational effect	1	2	3	4	5	Mean
Blood glucose sensor system	0	0	2	3	7	4.4
Physical activity sensor system	0	0	2	4	6	4.3
Nutrition habit registration system	0	2	0	4	6	4.2
Tips function	0	0	3	6	3	4.0

Questionnaire 3 - Agreement with effect of usage of the Few Touch application on behavior change in activities for self-management of diabetes (1: Totally disagree, 2: Disagree, 3: Neither / I don't know, 4: Agree, 5: Totally agree)

Behaviors / Agreement level on the effect of the application	1	2	3	4	5	Mean
Healthier nutrition habits	0	0	4	4	4	4.0
Being more physically active	0	0	4	4	4	4.0
Sufficiently frequent blood glucose measurement	0	1	4	6	1	3.6

Questionnaire 5 - Satisfaction level with knowledge about diabetes and with skills in diabetes management. (1: Very dissatisfied, 2: dissatisfied, 3: Neither / I don't know, 4: Satisfied, 5: Very satisfied)

Items and Time elapsed / satisfaction level	1	2	3	4	5	Mean
knowledge about diabetes						
Starting point (Meeting 1) [a]	0	0	1	9	1	4.0
6 months (Meeting 4)	0	0	1	10	1	4.0
1 year (Meeting 6)	0	0	1	10	1	4.0
Skills in conducting diabetes management						
Starting point (Meeting 1)	0	0	2	7	3	4.1
6 months (Meeting 4)	0	0	1	7	4	4.3
1 year (Meeting 6)	0	1	0	8	3	4.1

[a] One participant did not answer this question

Questionnaire 6 - Expected frequency of usage of the Few Touch application in future

Time elapsed / frequency of use	seldom	monthly	weekly	daily
6 months (Meeting 4)	0	0	2	10
1 year (Meeting 6)	0	1	4	7

Questionnaire 7 – Satisfaction level with the tips function (1: Very dissatisfied, 2: dissatisfied, 3: Neither / I don't know, 4: Satisfied, 5: Very satisfied)

Elapsed time / Satisfaction level	1	2	3	4	5	Mean
3-4 months (Meeting 3) [a]	0	1 [a]	1 [a]	5	6	4.1
6 months (Meeting 4) [29]	0	0	1	8	3	4.2
1 year (Meeting 6)	0	0	3	6	3	4.0

[a] One participant marked both 2 and 3 to this question. Therefore, for calculation of mean we counted the score by this participant as -0.5.

Questionnaire 8 - Distribution of the answers to questionnaire if the participants wish to have following elements in the Few Touch application. (1: Totally disagree, 2: Disagree, 3: Neither / I don't know, 4: Agree, 5: Totally agree) (adapted from [28])

Elements / Satisfaction level	1	2	3	4	5	Mean
A smaller step counter that is easier to wear	0	0	3	1	8	4.4
Tips that pop up on the mobile phone screen automatically	0	0	1	6	5	4.3
Automatic feedback based on measurements and personal medical data	0	0	2	7	3	4.1
Use of own mobile phone	0	1	2	4	5	4.1
Reminder for blood glucose measurement	0	0	4	4	4	4.0
Feedback delivery on mobile phone from healthcare professionals	0	2	1	4	5	4.0
Reminder for nutrition habit recording	0	0	4	5	3	3.9
Data transfer to general practitioner when the data is alarming	1	0	2	5	4	3.9
Platform of communication with patient peers for message exchange, data/goal sharing	0	3	2	3	4	3.7
Data transfer to general practitioner on regular basis	1	1	4	5	1	3.3

Questionnaire 9 - Answers to questions about whether the following issues have been improved in the course of the trial (“x” for yes)

Participant	Medication	Nutrition habits	Level of physical activity	Blood glucose control
P01		x	x	x
P02			x	x
P03		x		
P04		x	x	x
P05			x	
P06		x		x
P07				
P08			x	x
P09				
P10		x	x	
P11		x	x	x
P12		x	x	
Total	0	7	8	6
