# Supplementary material for: Long-Term Engagement With a Mobile Self-Management System for People With Type 2 Diabetes
Source: JMIR Mhealth Uhealth. 2013 Mar 27;1(1):e1. doi: 10.2196/mhealth.2432 (PMC4114413; doi:10.2196/mhealth.2432)
Supplement: Supplementary file 5 [file mhealth_v1i1e1_app5.pdf]

Multimedia Appendix 5 – Summary of prominent themes and codes with examples of quotes.

Theme: Learning oneself through use of the application	
Codes	Examples of quotes
Finding relationships between their self-management and blood glucose levels	“It shows that if I have eaten and exercised, and if I eat and don’t exercise, I get different [blood glucose] measures. So it shows simply how important it [physical activity] is.” (P01, at Meeting 4)
	“I saw the rise in BG over 12, 13 after the pneumonia.” (P04, at Meeting 4)
Constant use over time to get overview of their self-management activities and their blood glucose levels	“I see that blood sugar varies and I eat a little differently, so this here is an acknowledgement of that” (After P01 said that s/he found that his/her nutrition habits and physical activity were more or less the same every day.])“I don’t walk the same amount every day, so it [physical activity] varies a lot from day to day. Some days I walk a lot, but some other days I don’t have capacity to walk. This is easy to spot by looking at the tool [Diabetes Diary].” (P03, at Meeting 5)
	“I think it is good to get the graph and to be able to see how I lie on the slope [meaning ’how my blood glucose level changes over time’], and then I can reflect on what I ate.” (P10, at Meeting 4)
	“When it comes to the diabetes program, I would say that for me it has been a confirmation of whether or not I perform [self-management] more or less properly. It is clear that it [the application] does. I have not made any particular changes in either physical activities or nutrition habits, but when [the blood glucose level] is under seven [mmol/l], at six and down to five –sometimes eight or so every morning, then I think I have done it well.” (P09, Meeting 6)
Sporadic use in out-of-the ordinary situations	“It is so difficult when you are away. I was on a ferry trip from Bergen. And the food you got everywhere was absolutely not one for us. Nothing coarse [no coarse grain bread]. Even at the hospital here, I sit and wait for checkups and so forth. I thought I should take a salad, but even that salad has so much macaroni (laughter). … I was visited my sister in Spain. She has arthritis. She never talks about it. She gets irritated with me when I say ‘I cannot eat this because I have diabetes’. She gets angry (laughter). Oh my God, I need to be careful. … Then when we started driving out, I ask ‘how long it would be. Will the driving take long, so I must bring my food?’ ‘No-o-o’ (laughter). We drove up to the mountains, and so on. Then, what happened to me? I felt like clicking in my ear, but it was because it was very high. I had to go to a rest room and secretly measure so that she would not see it, and, I come staggering out, and look, it was 2.3. I was totally…. Oh my God, I just wondered how I would manage to get out of the rest room.” (P02, at Meeting 2)<br>“I use this [nutrition habit recording system] only when travelling, because I am [otherwise] very systematic person regarding foods” (P02, at Meeting 4)<br>“I use this [nutrition habit recording system] when travelling, because I tend to have hypo” (P02, at Meeting 6)
	“Yes, I use it[nutrition habit recording system]. I try to. (A researcher:

	"Even after nine months have passed?") Yes. (A researcher: "how do you manage to continue using it?") No, I just use what you installed in the mobile phone (laughter). It is easy for weekdays, because I know the pattern, this and this and this to eat. The worst is on weekends because it happens that I go out and have meals at different time. Then you take [the application], counts and summarize up. (A researcher: "Do you still get anything out of using it?") Oh yes." (P12 at Meeting 5)
Experimental use to examine how blood glucose level, self-management activities and quality of life are related	"Another thing is that as a person with diabetes, it is a dream to have a good sleep. It really is. I don't know how many experience that, but at least for me it is like that. (Another participant agreed.) So actually I used this [application] to find out what I can do for the best sleep: when I eat in the evening, when I walk, is it too late, right? Did I eat too late or early? It is to find out the right time to eat and to eat right meals. (Three other participants agreed: "totally right", "yes") … (P08 asked: "But you don't get the time at which you record food?") No, no it does not. But I know, I am not very good at recording foods, but I know what I ate in the evening and the time, and then when I take a measurement [in the evening], I register foods. So I can see like, when I slept best and how I did then. It [the application] is very well. In the last few months I used the system for that [purpose]." (P01 at Meeting 5)
Theme: Increased motivation for: using the application / self-management	
Codes	Examples of quotes
Through both goal achievement and learning oneself	"And when it is recorded – both blood sugar and step counts – and when I go to a meeting, the step counter has made me park the car as far as possible from the meeting – I even make a detour. Before, I parked as close as possible and walked the shortest distance. Now it is totally the opposite." (P01, at Meeting 4, continued from the quote introduced under "learning oneself through use of the application", Quoted in [53])
Through goal achievement	"As long as the step counter worked, I had it with me. It became like a little, a little competition with myself compared with the day before, at least [I must] come up to that level or a little more. So in that sense it worked well." (P12, at Meeting 6)
	"I think it [goal achievement] is very important. I got [system developer's name]'s step counter, and I believed that he set 8000 [as a goal for step counts per day]. I think it is fine to have 8000 [as a goal], because this I can manage [to walk], but not 10 [ten thousand]. Then I become sad, and think 'oh no, I cannot achieve the preset goal' "(P07, at Meeting 5)
Through learning oneself	" I had a period in which I changed medication from tablets to insulin. So I used it [the application] to find, eh because I had too high blood sugar, to find the right insulin dose for the different food items. So it was little tiring then. Then a whole lot started, like up and down and many high values and so on. So, but it was anyhow fine now because I am learning, how much insulin to use for this and this and so on. So I think it is nice, very good help tool" (P06, at Meeting 2)<br>"Yes, I use it [nutrition habit recording system]. It is fine to use. I have forgotten to record several times, but normally I record when I eat or

	after eating. Now I eat meals with a high content of carbohydrate less often. It is motivating. I think more about what I eat." (P06, at Meeting 3)
	"I think the tips are very good. I got a lot of motivation by reading. Quite a lot of tips, 80." (P04, at Meeting 3)
Theme: Consequence in terms of participants' self-management of diabetes	
Codes	Examples of quotes
Improvement in attitudes and behaviours	"I see very much in the graph. That is, in a word, to see, (pause) one can visually see it as it is. It is very important, and led me to discover that I had a problem [with treatment] in a word (other participants' laughter). I worked hard for seven hours in a wet field, standing [did physical work] and was very much stressed. I measured afterward. I ate only two wholemeal bread rolls that morning, and I could not get down to 6.0 [mmol/l]. It was 9.8, I remember that I saw 9.8 on the meter after I got totally exhausted. Then I understood that something was wrong. So, I am very glad [to use the application] basically. Now I am negotiating (laughter) with my doctor, 'what can we do?' (A researcher followed up the statements by asking: 'So did you increase the frequency of blood sugar measurements? Do you measure more often?') Yes, yes. Now I have almost used 100 strips." (P05, at Meeting 2)
	"I did not eat so much sweet food in the Christmas season. Instead, I ate a lot of fruits and a little bit of cake every now and then". (P03, at Meeting 3)
Sense of mastery over diabetes	"I have become more certain about how to tackle my diabetes with this [application]. I got more control without walking around thinking about it [the diabetes] all the time. I have it at the back of my mind, but it is not like before - that I was unsure and had a guilty conscience, which is what I don't have anymore." (P12, at Meeting 4)
	"I don't think about it [diabetes] so much anymore. I don't get the guilty conscience that I had before, because I think I can manage to control it much better now. But now it has been a special situation for me anyhow, during the [last] year I was sick, so it becomes a little special and the blood sugar has come down more. I hope that I don't become as lacking in energy as before. But I don't get a guilty conscience like before. This [the application] helps and supports me in keeping up, for the better " (P08, at Meeting 4)
Theme: Decrease in usage/leaving the application	
Codes	Examples of quotes
By gaining sense of mastery over diabetes and decreasing motivation for using the application	"To be honest I have been lazy and have not bothered myself to record on this here with food and drinks, because it shows that, I did a lot in the first week or 14 days, I tried to be rather aware. But … I always reached the goals that had been set, for example, high carb. drinks and such things, for there it was zero, because I don't drink soft drinks or beers or that type of things." (P09, at Meeting 2)
	"I have not used the application in the last four months since the summer

	holidays. But the HbA1c went down from the last time. I believe that I have gained a better understanding about myself, and now I don't have to use it daily as I did before. I was eager at the beginning. Even though I don't use it, I think over what I eat and things like that anyways." (P11, at Meeting 6)
	"I have not used it [the provided smartphone] as a mobile phone or Diabetes Diary in the last months. I have put it aside, because I was so bothered by it [the provided smartphone; P04 later mentioned that the smartphone had problems, for example that the phone hung up]. I used it very much in the beginning, though. In the last month when I was at the doctor, my HbA1c went down. It [the application] is useful for the people who are recently diagnosed." (P04, at Meeting 6)
	"I recorded all foods and such. I think it is incredibly good that one can [record nutrition habits], because it is me who can manage them [nutrition habits] all, basically. But I didn't measure [blood glucose levels] so much, because I thought that was a bit of a stress factor. As long as I can feel in my body how it works, I feel fine. But recording foods and training, I can get them under my control, then I can manage the other [blood glucose level]. So I like the application. Of course I did manage to do control measurements at least once or twice a week, but not every day. I did it at the beginning to see, though. It is at least good to have a graph afterwards" (P12, at Meeting 6)
